# Promoting water consumption among Dutch children: an evaluation of the social network intervention *Share H*_*2*_*O*

**DOI:** 10.1186/s12889-021-10161-9

**Published:** 2021-01-22

**Authors:** Crystal R. Smit, Rebecca N. H. de Leeuw, Kirsten E. Bevelander, William J. Burk, Thabo J. van Woudenberg, Laura Buijs, Moniek Buijzen

**Affiliations:** 1 Behavioural Science Institute, Radboud University, Nijmegen, The Netherlands; 2 Erasmus School of Social and Behavioural Sciences, Erasmus University Rotterdam, Rotterdam, The Netherlands; 3 Radboud Institute for Health Sciences, Radboud University and Medical Centre, Nijmegen, The Netherlands

**Keywords:** Children, Drinking water, Intervention, Motivation, Peer influence, Sugar-sweetened beverages

## Abstract

**Background:**

There is a need to develop and improve interventions promoting healthy drinking behaviors among children. A promising method could be to stimulate peer influence within children’s social networks. In the *Share H*_*2*_*O* social network intervention (SNI), peer influence was utilized by selecting a subset of influential children and training them as ‘influence agents’ to promote water consumption—as an alternative to SSBs. Previous research has mainly focused on the process of selecting influence agents. However, the process of motivating influence agents to promote the behavior has hardly received any research attention. Therefore, in the SNI *Share H*_*2*_*O* SNI, this motivation process was emphasized and grounded in the self-determination theory (SDT). This study evaluated the implementation of the *Share H*_*2*_*O* SNI, focusing on whether and how applying SDT-based techniques can motivate the influence agents and, indirectly, their peers.

**Methods:**

This study included data collected in the Netherlands from both the influence agents (*n* = 37) and the peers (*n* = 112) in the classroom networks of the influence agents.

Self-reported measurements assessed the influence agents’ enjoyment of the training, duration and perceived autonomy support during the training, and changes in their intrinsic motivation and water consumption before and after the start of the intervention. Changes in the peers’ intrinsic motivation, perceived social support, and social norms were measured before and after the start of the intervention.

**Results:**

The influence agents enjoyed the training, the duration was adequate, and perceived it as autonomy supportive. There was an increase in the influence agents’ intrinsic motivation to drink water and their actual water consumption. Providing personal meaningful rationales seemed to have motivated the influence agents. The intrinsic motivation and perceived descriptive norm of the peers remained stable. The peers reported an increase in their perceived social support and injunctive norm concerning water drinking after the intervention. Influence agents appeared to mainly use face-to-face strategies, such as modeling, talking to peers, and providing social support to promote the behavior.

**Conclusions:**

The current findings provided preliminary evidence of the promising effects of using SDT-based techniques in an SNI to motivate the influence agents and, indirectly, their peers.

**Trial registration:**

NTR, NL6905, Registered 9 January 2018, https://www.trialregister.nl/trial/6905

## Background

The prevalence of childhood overweight and obesity has increased at an alarming rate worldwide [1]. The increasing consumption of sugar-sweetened beverages (SSBs) has been identified as a major contributor to these rising levels [2]. The majority of children (61%) consume at least one SSBs on a given day with an average of 132.5 kcal/day [3]. Reducing the consumption of SSBs has proven to be an effective strategy to decrease weight gain in children [4]. In particular, replacing the consumption of SSBs with water seems to be a promising approach [5]. Unfortunately, data from several countries suggest that children’s daily water consumption is below recommended levels [6–9]. There is therefore a need for interventions aimed at promoting water consumption among children and thus reducing their SSBs consumption. However, recent evidence identifies that previous interventions have only had small positive effects on the water and SSB consumption of children [10].

A promising method for interventions may be to incorporate the influence of the social environment in order to promote water consumption among children. There is sufficient evidence that the social environment strongly influences the consumption behavior of children [11–14]. As children grow older, their susceptibility to peers increases, peaking during early adolescence [15]. Extensive systematic reviews have therefore also shown that peers play an important role in children’s food choice and intake [16, 17]. For example, peers can establish a social guideline (i.e., social norm) on food choice and intake which can be followed by others [18]. In social modelling studies, children also appear to directly adjust their intake to that of their table companions [13]. Children also tend to consume more food when they are in the presence of several peers [19]. Despite this important role of peers, until recently peers have been relatively overlooked in many interventions aimed at the consumption of water and SSBs for children [10]. An intervention approach that utilizes peer influence to address health-related behaviors is the so-called “social network interventions (SNIs)” [20, 21].

In recent years, there has been a growing interest in the use of social network interventions in the field of public health [22–27]. At the heart of this approach lies the diffusion of innovations theory, which conceptualizes how individuals can act as change agents to informally diffuse new beliefs and behaviors in a social network [28]. Based on this premise, interventionists select a subset of individuals as influence agents to initiate the diffusion of the target health behaviors in their social network [29]. Accordingly, in the SNI called *Share H*_*2*_*O*, children were selected as influence agents and trained to promote water consumption—as an alternative to sugar-sweetened beverages (SSBs)—among their peers [25, 30, 31]. As reported elsewhere [31], the *Share H*_*2*_*O* intervention was effective in increasing water drinking and reducing SSBs, with the effectiveness on water drinking depending on the prevailing social norms in the classrooms. In particular, children with higher perceived descriptive norms and lower perceived injunctive norms reported an increase in their water drinking. The study reported here evaluates the implementation of the *Share H*_*2*_*O* SNI.

Previous research has mainly focused on the process of *selecting* the most successful influence agents by investigating the best peer nomination questions and selection criteria to identify them. However, despite the underlying premise of SNIs that the selected influence agents diffuse the desired behavior in their network, the process of *motivating* the influence agents to do so has hardly received any research attention [24, 25]. To fill this gap, the current study focuses on the process of *motivating* the influence agents in SNIs to diffuse the target behavior in their social network. The evaluation followed the theoretical framework that guided the design of the *Share H*_*2*_*O* training in order to motivate the selected influence agents and, indirectly, their peers.

The *Share H*_*2*_*O* training was grounded in self-determination theory (SDT), a prominent theory of human motivation [32, 33]. Research on SDT has amply demonstrated that intrinsic motivation, the most autonomous kind of motivation, plays a central role in facilitating health behavioral change and its maintenance [34]. Intrinsic motivation refers to doing something because it is inherently interesting or enjoyable [33, 35]. Individuals who are intrinsically motivated are more likely to adopt and maintain health-related behaviors [36], such as drinking more water [37]. According to SDT, being intrinsically motivated depends on the satisfaction of three basic psychological needs: autonomy (feeling that one is responsible and has choice), competence (feeling that one is capable and effective), and relatedness (feeling respected and close to others [33, 38]). These three needs can be satisfied by creating an autonomy-supportive climate, involving SDT-based techniques, such as providing meaningful rationales, choice, and support, and encouraging self-initiative [38, 39]. Therefore, in order to optimally motivate the influence agents and, indirectly, their peers, the *Share H*_*2*_*O* training was developed to foster an autonomy-supportive climate. This was done by facilitating their basic psychological needs by applying SDT-based techniques in the training.

### Research aims of the current study

The focus of this study is to evaluate the implementation of the *Share H*_*2*_*O* intervention, in particular whether and how applying SDT-based techniques can motivate the influence agents and, indirectly, their peers. We used reports of both the influence agents and the peers in the classroom networks of the influence agents. Based on the framework guiding the *Share H*_*2*_*O* intervention, we addressed three specific research aims. The first aim was to evaluate the influence agents’ general experiences with the training by assessing their ratings of their enjoyment of the training, the duration, and perceived autonomy support during the training. The second aim was to assess whether the training motivated the influence agents to drink more water themselves by examining changes in the influence agents’ intrinsic motivation and water consumption before and after the start of the intervention. The third aim was to examine whether the influence agents were successful in motivating their peers by investigating changes in the peers’ intrinsic motivation, perceived social support, and perceived social norms before and after the start of the intervention.

## Methods

### Design

This study was integrated into the *Share H*_*2*_*O* intervention effectiveness study [31], which was part of the second data collection phase of the *MyMovez* research program (see [40] for a detailed description of the *MyMovez* program). The study reported on data collected from both the selected influence agents and their peers. The required sample size for the *Share H*_*2*_*O* effectiveness study was based on the previous pilot study [25], in which a small effect on water and SSB consumption was found with 210 children in the intervention and control condition. To calculate the sample for the effectiveness study [31], this number was multiplied by 1.5 to add the third group (i.e., the active control), resulting in a minimum number of 315 children across the three groups. Ethical approval was obtained from the Ethics Committee of the Faculty of Social Sciences at Radboud University (ECSW2014–100614-222) and the ethical review board from the European Research Council (617253). The design of the *Share H*_*2*_*O* SNI was preregistered at the Netherlands Trial Register (NL6905).

### Procedure

Both suburban and urban schools throughout the Netherlands were invited to participate via an email to the school principal. Only primary or secondary schools following a regular education program and with classes between the 4th and 7th grade (i.e., students aged 9 to 13 years) were invited to participate in the *MyMovez* project. The project focused on this age group because it is important that children learn healthy intake behaviors at an early age since the increase in overweight and obesity is the steepest around the ages of 16 to 20 years [41] and intake habits and preferences developed in childhood can persist into adulthood [42]. After obtaining written permission from the school principals, an information letter was distributed to the children and their parents. In addition, pitches were delivered in school classes to recruit participants. Given the age of the participants (< 16 years), written informed consent was obtained from a parent or legal guardian as well as the children themselves. Subsequently, the participating schools were randomly assigned to one of the five conditions of the *MyMovez* project (see [27, 31] for a detailed description of the conditions). The current study sample included the five (sub) urban primary schools (i.e., eight classes from grades 4–6) assigned to the condition exposed to the *Share H*_*2*_*O* SNI.

For the overall *Share H*_*2*_*O* SNI, data were collected at baseline (T1; February–March 2018) immediately after the start of the intervention (T2; April–May 2018), and during a follow-up 4 weeks later (T3; June–July 2018). The evaluation measurements of the current study were collected at T1 and T2 only. At each assessment, children received a smartphone with a preinstalled research application and a wrist-worn accelerometer for 7 days [40, 43]. Via the research application, children received daily questionnaires and were able to use a social media platform (*Social Buzz*), create a personalized avatar, and play a puzzle game. In the *Social Buzz*, children could chat, share pictures, and short videos with their peers through the social media platform integrated in the research application.

### The Share H_2_O SNI

Briefly, the SNI comprised of (1) identifying and selecting the influence agents and (2) training the influence agents, followed by an informal follow-up a week later. The influence agents were identified through peer nominations. Children nominated the peers on four sociometric nomination questions (“Whom do you ask for advice?”; “Who in your classroom are leaders or take the lead often?”; “Whom do you want to be like?”; and “With whom do you talk about what you drink?” [44]). The selection criteria for the influence agents were those from each participating classroom who were most often nominated by their peers on all items combined. To ensure gender balance in relation to the composition of the classrooms, 15% of the boys and 15% of the girls with the most nominations were selected as influence agents. This resulted in an average of five children (range 3–6 children; *SD* = 1.06) per participating classroom being trained as influence agents [31].

The influence agents’ training lasted 1 hour and took place at school, led by research assistants who worked in pairs. The research assistants were trained (≈ 8 h) by skilled researchers who had ample expertise in conducting research with children at schools and with an autonomy-supportive approach to working with children. The research assistants all had a background in pedagogical sciences, in which they studied the development of children and adolescents. To ensure that each training session in the intervention classroom was conducted in a similar fashion, the principal trainer accompanied each research assistant on their first training session and provided them with a guideline to facilitate the delivery of the training. This guideline contained information about *Share H*_*2*_*O* in general, the theoretical principles of the intervention approach and training, and a detailed script to implement each technique in the training. In addition, the research assistants were in constant contact with the principal trainer, and interim evaluations were performed after each training was given.

As described above, the *Share H*_*2*_*O* training was grounded in self-determination theory and refined with input from children and research experts, and thereafter extensively tested in two pilot studies [25, 30]. One week after the training, a half-hour follow-up training session took place at school. This follow-up session provided the research assistants with the opportunity to offer visible support to the influence agents, resolve any problems experienced by the influence agents, and refresh the core topics discussed in the initial training. In the following sections, we describe how the training implemented SDT-based techniques to motivate influence agents to drink more water and support them in motivating their peers to drink water (a detailed overview of all training materials is available upon request).

#### Motivating influence agents to drink more water themselves

The first part of the *Share H*_*2*_*O* training focused on motivating the influence agents to increase their own water consumption. To achieve this, we implemented two SDT-based techniques in the training: providing meaningful rationales for drinking water and prompting the influence agents to self-initiate the target behavior [35, 45, 46].

**Fig. 1:**
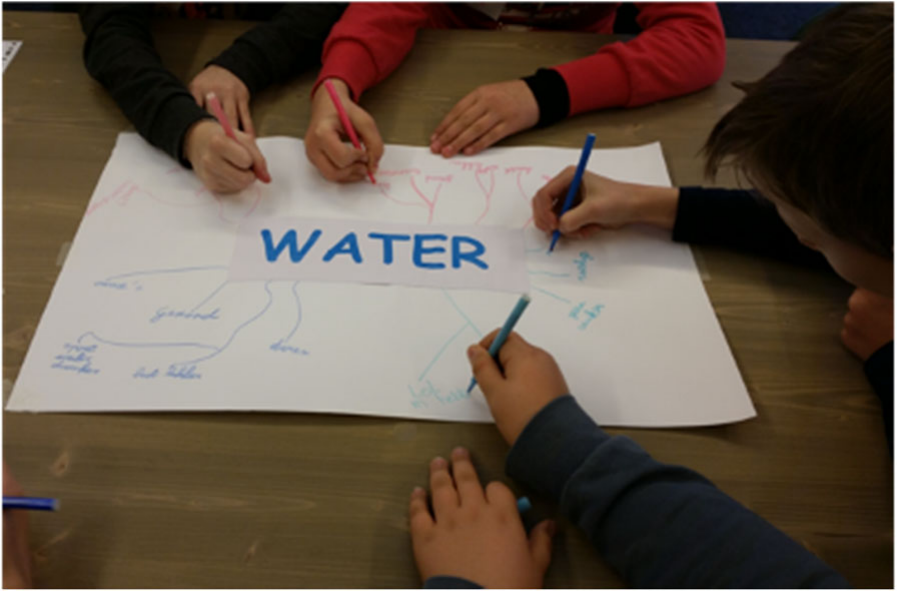
Influence agents working together on a word web about the benefits of drinking water. This picture was taking by the principal researcher during a training.

The technique of providing meaningful rationales for drinking water highlights and reinforces personally meaningful and valuable rationales that could form the basis for intrinsic motivation [35, 45, 46]. Research has shown that even with a boring task, meaningful rationales can lead to internalization [47]. This technique was implemented in the training by discussing the benefits of drinking water. First, all influence agents were asked to brainstorm about the benefits by working together on a word web (see Fig. 1). This allowed them to learn meaningful and valuable benefits from their peers—to which children at this age are highly susceptible [15]. Subsequently, the trainers supplemented these benefits through an interactive presentation which included a range of health (e.g., “Water does not contain sugar” and “Water is the best thirst quencher”) and environmental benefits (e.g., “Drinking water is good for the animals and the nature”) for drinking water. The presentation also included quiz questions in which the influence agents learned, for example, that the recommendation is to drink 1.5 l of water per day. All the benefits in this presentation were based on short-term outcomes (e.g., “Drinking water makes your skin beautiful” and “Drinking water ensures that you can concentrate better”) because these are considered more motivating than long-term consequences [48].

**Fig. 2:**
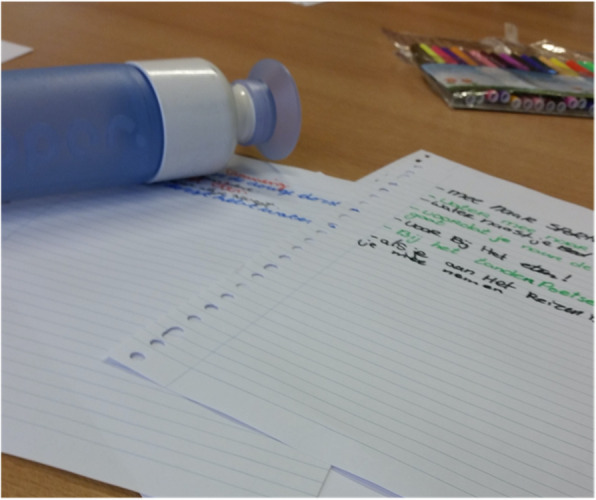
An example of a sheet containing the self-generated arguments of the influence agents. This picture was taking by the principal researcher during a training.

The technique of encouraging self-initiation of drinking water involves prompting individuals to initiate the target behavior themselves, which provides them with an opportunity to learn and develop the associated skills, all of which support their intrinsic motivation [35, 45, 46]. Hence, after discussing the benefits of drinking water in the training, the influence agents were encouraged to drink more water themselves through the use of self-persuasion [49]. This involved placing them in a situation where they had to persuade themselves to drink more water [50, 51]. More specifically, the influence agents were asked to generate their own arguments that indicate how they could drink more water in order to persuade themselves to do so (see Fig. 2).

#### Supporting influence agents in motivating their peers

The second part of the training focused on supporting the influence agents in their task of motivating their peers to drink more water. For this purpose, two SDT-based techniques were used in the training: allowing the influence agents to choose how to motivate their peers and providing them with the skills to do so [35, 45, 46].

The technique of providing choice promotes personal input and ownership of the behavioral change [46], which facilitates individuals’ need for autonomy [38, 45, 46]. Ample research suggests that individuals are more intrinsically motivated to perform the target behavior when provided with choices [52–54]. In order to support the influence agents in motivating their peers, influence agents were encouraged in the training to choose how exactly they wished to motivate their peers. Therefore, the influence agents were asked to think and decide for themselves concerning how to promote water drinking and were facilitated in sharing their devised ideas with their peers.

**Fig. 3:**
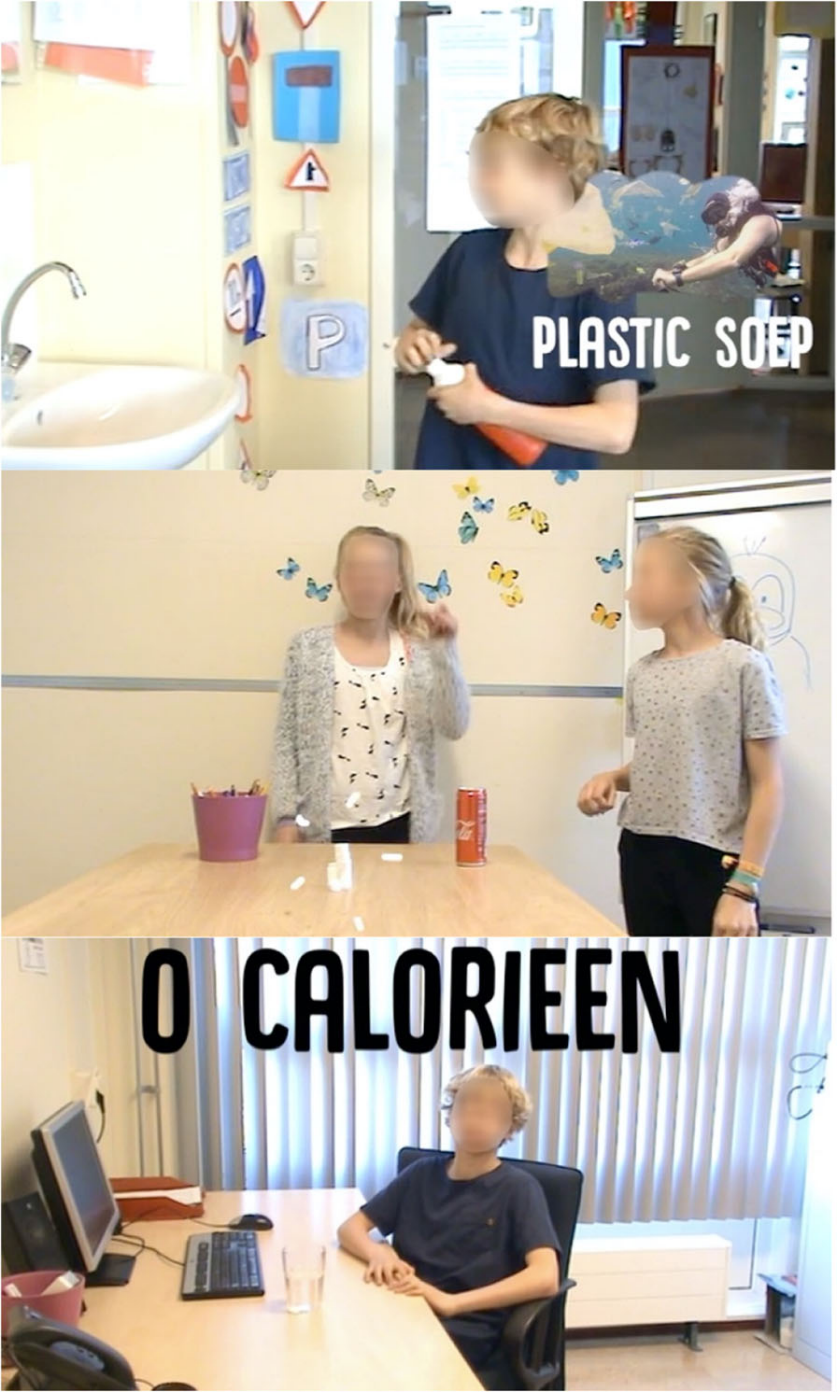
Screenshots of the short videos that the influence agents could spread among their peers. The short videos were made by the researchers. The first screenshot is from a scene where a child talks about the environmental benefits of drinking water. In this scene, the child explained that if more people drink water, less plastic is made in the factories because you can drink water from the tap. This allows less plastic to end up in the plastic soup in the North Pacific Ocean. The short videos also included other benefits of drinking water, including that water contains no sugar and has zero calories. All these benefits correspond to the ones discussed in the presentation during the training and were suggested by the children themselves

The technique of providing the influence agents with skills on how to motivate peers included providing information on how to perform the target behavior and promoting the feeling of competence in the behavior [35, 45, 46]. Therefore, in the training, through possible scenarios, it was discussed how and when they could promote water drinking among their peers to provide them the skills to do so. A possible water-promoting strategy discussed in these scenarios was setting a good example by drinking water themselves. Research has shown that children tend to model the intake behavior of their peers [13]. In addition, it was also discussed that they could promote water drinking through informal communication [28], for example, by talking about water drinking at school or sending messages and short videos about it (see Fig. 3) on *Social Buzz* to their peers. Subsequently, they brainstormed together about potential barriers they might encounter and how to overcome them. Finally, the influence agents were continuously supported by the researchers in motivating their peers, which corresponds to their need for relatedness [35, 45, 46].

### Measurements

The sections below describe the evaluation measurements used to collect quantitative (close-ended) and qualitative (open-ended) data from both the influence agents and the peers. Table 1 presents an overview of the quantitative measurements.

**Table 1:** Quantitative evaluation measures of the influence agents and their peers at T1 and/or T2

Measure name	Item(s)	Response options	Data sample	Time point
Enjoyment of the training	Did you like the training related to drinking water?	No, not at all No, not really Yes, a little bit Yes, a lot	Influence agents	T2
Duration of the training	What did you think of the duration of the training related to drinking water?	Too short Short Neither too short nor too long Long Too long	Influence agents	T2
Perceived autonomy support	I had the feeling that the researcher gave me choices. I felt understood by the researcher. The researcher showed that she had confidence in me to stimulate water drinking. The researcher encouraged me to ask questions. The researchers listened to how I wanted to stimulate water drinking. The researcher tried to understand my ideas before she herself came up with other ideas.	No, not at all No, not really Yes, a little bit Yes, a lot	Influence agents	T2
Intrinsic motivation	I drink water because … … I like it … I enjoy it … I think it is pleasant	No, certainly not No, I do not think so No, possibly not Yes, possibly Yes, I think so Yes, certainly	Influence agents and peers	T1 and T2
Water consumption	How many glasses of water did you drink yesterday?	Zero glasses per day One glass per day Two glasses per day Three glasses per day Four glasses per day Five glasses per day Six glasses per day Seven or more glasses per day	Influence agents	T1 and T2
SSB consumption	How many glasses of sweetened fruit juice did you drink yesterday? How many glasses of lemonade made of sugar syrup and water did you drink yesterday? How many glasses of soda did you drink yesterday? How many glasses of energy drink did you drink yesterday? How many glasses of sport drink did you drink yesterday?	Zero glasses per day One glass per day Two glasses per day Three glasses per day Four glasses per day Five glasses per day Six glasses per day Seven or more glasses per day	Influence agents	T1 and T2
Perceived social support	How often do your peers... … complement you on drinking water? … participate in drinking water with you? … remind you to drink water? … offer to drink water with you?	Never Rarely Sometimes Often Very often Always	Peers	T1 and T2
Descriptive norms	How often do your classmates drink water?	Never Rarely Sometimes Often Very often Always	Peers	T1 and T2
Injunctive norms	Do you experience that your classmates think you should drink water?	No, certainly not No, I do not think so No, possibly not Yes, possibly Yes, I think so Yes, certainly	Peers	T1 and T2
Water-promoting strategies	How often did you drink water when your classmates were with you? How often did you talk with your classmates about drinking water at school or home? How often did you talk with your classmates about drinking water on the social media platform (*Social Buzz*)? How often did you send videos about water drinking to your classmates on the social media platform (*Social Buzz*)?	Never Rarely Sometimes Often Very often Always	Influence agents	T2

*Note.* T1 = baseline; T2 = immediately after the start of the intervention

#### General experiences with the training

The influence agents’ *enjoyment of the training* was assessed using a 4-point scale ranging from 1 = “no, not at all” to 4 = “yes, a lot”, adapted from the level of enjoyment measure reported by Sebire et al. [55], and with open-ended responses about which parts of the training they enjoyed the most and least. Their experiences with the duration of the training were assessed using a 5-point scale ranging from 1 = “too short” to 5 = “too long”. The extent to which the influence agents’ *perceived autonomy support* during the training was assessed using the short form (six-items; see Table 1) of the Learning Climate Questionnaire [56], with response options ranging from 1 = “no, not at all” to 4 = “yes, a lot”.

#### Motivating influence agents to drink more water themselves

To evaluate whether the training motivated the influence agents, we assessed their intrinsic motivation and their water and SSB consumption. The influence agents’ *intrinsic motivation* to drink water was measured at T1 and T2, using three items (see Table 1) adapted from a scale based on exercising [37, 57], with a 6-point response scale ranging from 1 = “no, certainly not” to 6 = “yes, certainly”. A total score for intrinsic motivation was constructed by averaging the three items. To assess *water consumption* at T1 and T2, the influence agents indicated on three different days (i.e., every other day during each assessment) on an 8-point scale ranging from 0 = “zero glasses per day” to “7 = seven or more glasses per day” how much water they had drunk the day before. A total score for water consumption was constructed by averaging the influence agents’ reported consumption over the 3 days. To assess the influence agents’ *SSB consumption* they had to indicate on three different days (i.e., every other day during each assessment) how much sweetened fruit juice, lemonade (based on sugar syrup), soda, energy, and sports drinks they had drunk the day before [58]. The same response scale as with water consumption was used. To assist them in recognizing each of these types of beverages, examples of frequently consumed beverages were included for each item. A total score for SSB consumption was constructed by averaging the influence agents’ reported consumption on these five items over the 3 days. The influence agents also provided responses to several open-ended questions concerning their experiences with the training, which were used to evaluate the SDT-based techniques that were implemented to motivate them.

#### Supporting influence agents in motivating their peers to drink water

To evaluate whether the training supported the influence agents in optimally motivating their peers, we assessed their peers’ intrinsic motivation, perceived social support, and social norms regarding water drinking. The *intrinsic motivation* of the peers was measured at T1 and T2 with the same three items (see Table 1) as with the influence agents [37, 57]. A total score for the peers’ intrinsic motivation was constructed by averaging the three items, which demonstrated adequate internal consistency (Cronbach’s α_T1_ = .83; Cronbach’s α_T2_ = .87). Their *perceived social support* to drink water was measured at T1 and T2, using four items derived from a broader questionnaire on healthy behaviors [59], each rated on a 6-point scale ranging from 1 = “never” to 6 = “always”. A total score for perceived social support was constructed by averaging the four items, which demonstrated adequate internal consistency (Cronbach’s α_T1_ = .79; Cronbach’s α_T2_ = .86). The peers’ perceived social norm was assessed at T1 and T2, based on their beliefs about how often one’s peers drink water (i.e., *descriptive norm*; response options ranged from 1 = “never” to 6 = “always”) and their beliefs about the approval of one’s peer regarding drinking water (i.e., *injunctive norm*; response options ranged from 1 = “no, certainly not” to 6 = “yes, certainly” [37].

To evaluate the SDT-based techniques that were implemented in the training to support the influence agents in motivating their peers, we measured on a 6-point scale (ranging from 1 = “never” to 6 = “always”) whether the influence agents applied the water-promoting strategies discussed in the training: (1) *drinking water themselves*, (2) *talking about water at school or home*, (3) *talking* and (4) *forwarding short videos about water on a social media platform (Social Buzz)*. The influence agents also provided responses to several open-ended questions concerning the strategies they implemented to motivate their peers to drink more water and their experiences therein.

### Statistical analyses

All data were analyzed using SPSS version 25 (SPSS, Inc., Chicago, IL, US). Significance was set at *p* < .05. For the close-ended (quantitative) data related to the first and third research aim, we computed both means (*M*) and standard deviations (*SD*) for the general experience measurements (i.e., influence agents’ enjoyment and perceived autonomy support) and water-promoting strategies, as well as the percentage (%) of influence agents with positive (score of 3 or higher) versus negative responses (score of 2 or lower) on these measurements. To analyze the quantitative data associated with the second and third aim, a series of paired sample *t*-tests were conducted to examine changes before and after the training in the influence agents’ intrinsic motivation, and SSB and water consumption (second aim); and their peers’ intrinsic motivation, perceived social support, and perceived social norms (third aim). It should be noted that although the data fail to meet the assumptions of normality, the paired sample *t*-test was nevertheless chosen over the customary Wilcoxon signed-rank (nonparametric) test due to the findings supporting its application in small samples involving non-normal distributions, and/or ordinal data [60]. Table 2 presents the descriptive statistics of these quantitative measurements.

**Table 2:** Descriptive statistics of the evaluation measures of the influence agents and their peers

	T1			T2	
	*M (SD)*	Range	*% (n)*	*M (SD)*	Range
General experiences with the training					
Enjoyment of the training	3.66 (.55)	1–4	84% (31)		
Duration of the training	3.09 (.59)	1–5	84% (27)		
Perceived autonomy-support	3.54 (.38)	1–4	97% (30)		
Motivating influence agents to drink more water themselves					
Intrinsic motivation	4.48 (1.71)	1–6		4.93 (1.20)^b^	1.5–6
Water consumption	2.82 (1.97)	0–7		3.51 (2.02)^b^	0–7
SSBs consumption	.75 (.73)	0–7		.57 (.80)	0–3.6
Supporting the influence agents in motivating their peers					
Intrinsic motivation	4.61 (1.33)	1–6		4.43 (1.48)	1–6
Perceived social support	1.91 (.92)	1–6		2.16 (1.13)^b^	1–6
Descriptive norms	3.64 (.94)	1–6		3.66 (1.18)	1–6
Injunctive norms	3.73 (1.63)	1–6		3.33 (1.80)	1–6
Drinking water themselves	4.10 (1.18)	1–6	95% (20)		
Talking about water at school or home	3.05 (.97)	1–6	76% (16)		
Talking about water in the social media platform	2.05 (1.32)	1–6	27% (6)		
Forwarding short videos about water in the social media platform	1.95 (1.02)	1–6	24% (5)		

*Note.* Percentage (%) refers to the number of influence agents with a response score of ≥3; ^b^Findings from *t*-tests indicated significant differences over time; T1 = baseline; T2 = immediately after the start of the intervention

A content analysis was performed on the open-ended (qualitative) data related to the research goals. First, the primary researcher openly coded the open-ended responses to compile the categories, and afterwards, a second researcher coded the responses using the compiled categories. The responses of the influence agents related to their experiences with the training were classified based on the techniques implemented in the training. The influence agents’ responses concerning how they motivated their peers were classified based on whether or not they had set a good example themselves (i.e., modelling [13]), talked about water and its benefits [28], and/or had offered social support [59]. The Krippendorff’s alpha test was used to estimate the interrater reliability between the two coders [61]. The interrater reliability ranged from acceptable to good (Krippendorff’s alpha ranged between α = .77 and α = 1.00). Finally, the percentages of influence agents in the compiled categories were reported. Additional analyses (i.e., Pearson’s correlations) were performed to explore the effect of the training on the changes in intrinsic motivation, social support, and perceived social norms for different demographic variables of the peers (i.e., sex, grade level, and family affluence). The interindividual change score between the two assessments of the measurements were included as a change variable in the correlation analyses.

## Results

### Demographic characteristics

The sample of the current study consisted of 37 influence agents and 112 peers in the classroom networks of these influence agents. There were on average five influence agents per intervention class, aged between 9 and 13 years (*M* = 10.95, *SD* = .94). Their peers were between 9 and 14 years of age (*M* = 10.84, *SD* = 1.04). The majority of the influence agents and their peers came from high-affluence families (71.4% of influence agents and 69.4% of peers) [62].

### General experiences with the training

The majority (84%) of the influence agents responded that they had enjoyed the training (scoring ≥3; see Table 2). Only 9% of the influence agents made a negative remark about the training; they indicated that they found the plastic soup (i.e., the environmental impact of drinking SSBs compared to tap water) sad and found it difficult to accept that they should persuade others. Most of the influence agents (84%; see Table 2) indicated that the training duration was adequate; that is, they thought that it was neither too short nor too long. Only 3% of the influence agents indicated that the training was too short. Almost all (97%; see Table 2) influence agents perceived the training as being autonomy supportive. The separate items of the perceived autonomy-support measure revealed that the influence agents experienced that the trainers had made efforts to provide choice, to encourage them to ask questions, to listen and understand them, and to show confidence in their ability (percentages ranging from 68 to 84% of the influence agents). This indicates that the influence agents experienced support for autonomy, competence, and relatedness during the training.

### Motivating influence agents to drink more water themselves

Regarding the training process of motivating the influence agents, the influence agents on average reported significantly higher intrinsic motivation to drink water after the training as compared to before the training, *t*(26) = − 2.31, *p* = .029 (see Table 2), with 74% of the influence agents showing an increase. In addition to higher intrinsic motivation, the influence agents also reported drinking marginally significantly more water after the training compared to before the training, *t*(26) = − 1.89, *p* = .070 (see Table 2), with 67% showing an increase. The influence agents did not drink significantly fewer SSBs after the training as compared to before the training, *t*(26) = .88, *p* = .385 (see Table 2); however, about half (52%) of the influence agents did show a decrease.

The open-ended responses of the influence agents suggest that the technique of providing meaningful rationales motivated the influence agents to drink more water themselves. Specifically, most (47%) of the influence agents indicated that the word web in combination with the interactive presentation—in which the meaningful rationales to drink water were discussed—were the most enjoyable aspects of the training:

*“I liked the presentation the most [about the training].”*

*Girl, 10 years old*

*“I liked the most [about the training] that you can get handsome for free from drinking water and that you can get beautiful teeth.”*

*Boy, 12 years old*

*“The interactive presentation, for example, guessing how many sugar cubes there are in a 250 ml coca cola can.”*

*Boy, 10 years old*

Interestingly, these open-ended responses also revealed that some (13%) influence agents indicated that the most enjoyable aspect of the training was that they had to secretly encourage their peers to drink more water and thus were together part of a “secret mission”:

*“The fact that it [motivating their peers] had to stay a secret and I am part of a kind of spy-group.”*

*Boy, 12 years old*

### Supporting influence agents in motivating their peers

Regarding the training process of supporting influence agents in motivating their peers to drink more water, their peers’ intrinsic motivation remained stable. More specifically, after exposure to the intervention, the mean score of their intrinsic motivation to drink water was not significantly higher compared to before the intervention, *t*(91) = 1.38 *p* = .171 (see Table 2). Similarly, there were no changes in the mean for descriptive norms following the intervention, *t*(86) = .17, *p* = .867 (see Table 2), indicating that they did not perceive that their peers drank more water. The peers reported a marginal significant increase in their injunctive norm following the intervention, *t*(93) = 1.95, *p* = .054 (see Table 2), which implies that there is a trend showing that they perceived that their peers thought they should drink more water. The targeted peers also reported significantly higher social support to drink water after being exposed to the intervention compared to before the intervention, *t*(87) = − 2.34, *p* = .021 (see Table 2), meaning that they perceived that their peers more often complimented, reminded, offered, and participated in drinking water with them.

Related to this, the responses of the influence agents revealed that they used various strategies to promote water drinking among their peers. Regarding the water-promoting strategies discussed in the training, influence agents’ responses showed that they most often used face-to-face strategies to motivate their peers to drink water. Specifically, 95% (scoring ≥3; see Table 2) of the influence agents indicated that they had drunk water in front of their peers, and 76% (see Table 2) indicated that they had talked to their peers about drinking water at school or home in order to motivate them to drink water. Their open-ended responses about how they promoted water suggested that they often (34%) used the meaningful rationales and benefits that were discussed in the training:

*“Drink water. It is a good thirst quencher.”*

*Boy, 11 years old*

*“Water makes you perform better and can make you smart, so no more sugar-sweetened beverages but only water.”*

*Boy, 11 years old*

*“Saying water is healthy, you should actually drink it [water] more.”*

*Girl, 10 years old*

Twenty-seven percent (see Table 2) of the influence agents indicated that they had used the social media platform on the research application to talk to their peers about water drinking, and 24% (see Table 2) had forwarded the short videos about drinking water to their peers. The open-ended responses suggested that the influence agents not only motivated their peers by using the strategies discussed in the training, but based on the autonomy-supportive climate during the training, they themselves also devised ways to promote water. For example, some influence agents promoted water drinking by supporting their peers in drinking more water (19%), starting a challenge (3%), simply telling them that they had to drink water (3%), or promising rewards when they drank water (3%):

*“I asked in class if I had to fill their water bottles and mentioned the benefits of drinking water.”*

*Boy, 11 years old*

*“Can I fill your cup with water?”*

*Girl, 11 years old*

*“We made it into a challenge, and then we noticed that many children started bringing water to school to put on their table in class.”*

*Girl, 10 years old*

*“Said to them [their peers], you have to take a bottle to school on Wednesday.”*

*Boy, 11 years old*

*“I promised awesome rewards when they [their peers] would drink more water.”*

*Girl, 10 years old*

The open-ended responses of the influence agents suggested that the training had succeeded in providing some of them with the skills to promote water drinking among their peers. These influence agents namely indicated that they experienced that motivating their peers had gone well and that their peers reacted positively:

*“Went well, [name] immediately drank from my bottle of water.”*

*Girl, 11 years old*

*“They said yes, I am going to do it [drink water].”*

*Boy, 11 years old*

*“They said things like ‘Yes, you are absolutely right. Thanks for the tip!’”*

*Girl, 10 years old*

However, some influence agents also experienced that motivating their peers to drink water had gone less well. For example, they indicated that they mainly promoted water drinking in their family circle instead of among their peers. Others thought they had not sufficiently motivated their peers and also indicated that the next time they should be more concerned with motivating their peers. In addition, some also found it difficult to encourage their peers to drink more water:

*“I mainly tried it [motivating others to drink water] at home.”*

*Girl, 12 years old*

*“It [motivating others to drink water] went well, but I have not done it often.”*

*Girl, 11 years old*

*“Motivate my peers more often.”*

*Girl, 12 years old*

### Additional exploratory analyses

To scrutinize the effect of the training on the changes in the peers’ intrinsic motivation, social support, and perceived social norms, we also explored for which peers the *Share H*_*2*_*O* training specifically had caused a greater change. Pearson’s correlation analyses (see Table 3) revealed a significant negative relation between sex and changes in social support (*r* = −.26, *p* = .013), indicating that boys had a greater change in social support than girls. There was a significant positive relation between grade level and changes in intrinsic motivation (*r* = .22, *p* = .034) and injunctive norm (*r* = .19, *p* = .078), and a marginal significant positive relation between grade level and changes in social support (*r* = .26, *p* = .011). This indicates that children in higher grades had a greater change in intrinsic motivation, injunctive norm, and social support. There was no significant relation for family affluence.

**Table 3:** Correlations between the change variables and peers’ demographics

	Sex	Grade level	Family affluence
Changes in peers’ intrinsic motivation	.07	.22*	−.17
Changes in peers’ social support	−.26*	.19†	.17
Changes in peers’ injunctive norms	−.08	.26*	.17
Changes in peers’ descriptive norms	−.07	.08	−.12

*Note.* † *p* < .10, * *p* < .05, ** *p* < .01

## Discussion

This study is the first to investigate the process of motivating influence agents to diffuse the target behavior among their peers when implementing an SNI, in particular, whether and how applying SDT-based techniques can motivate influence agents and, indirectly, their peers. Diving deeper into this motivational approach and its application in SNIs provides insights that are valuable for both future research and interventions. The findings of this study are discussed below following the three research aims.

### General experiences with the training

In general, the findings showed that the influence agents had enjoyed the *Share H*_*2*_*O* training, found the duration adequate, and experienced it as autonomy supportive. The latter is highly important because an approach is only truly autonomy supportive if the intended individuals actually experience it this manner and not when the trainers alone think they were autonomy supportive. Previous work has shown that, for example, parents tend to overestimate how autonomy supportive they are towards their children [63]. Our findings suggest that an SNI based on the SDT approach can foster an autonomy-supportive climate, which may have enhanced the influence agents’ intrinsic motivation to perform the target behavior.

This approach also fits in the Dutch educational system—and probably in most Western countries—as schools are quite autonomous and have educational freedom [64]. Furthermore, an autonomy-supportive learning style is stimulated in the schools where children are granted responsibility and freedom in their learning process [65, 66]. This approach could also be integrated into existing dietary intake programs at schools, such as the national approach called *Gezonde School [Healthy School*] that supports schools in promoting a healthy lifestyle for their students . Based on our findings, schools could use an autonomy-supportive approach to motivate healthy dietary behaviors among their students.

### Motivating the influence agents to drink water themselves

Implementing the SDT-based techniques in the training appeared to have increased the influence agents’ intrinsic motivation to drink water and their actual water consumption. Providing meaningful rationales [35, 45, 46] especially appears to have motivated the influence agents, as they indicated that they enjoyed this part of the training the most and used the provided rationales to promote water drinking among their peers. Apparently, the provided short-term rationales [48] were meaningful for the influence agents. In addition, a self-persuasion technique [49] was also implemented in the training to encourage the influence agents to drink more water. Even though there was no evidence from the open-ended responses for the effectiveness of this technique, it does not necessarily mean it did not have an effect on motivating the influence agents, as most of them did increase their water consumption following the training.

### Supporting the influence agents in motivating their peers

Providing the influence agents with the skills to promote the target behavior, by discussing possible water-promoting techniques with them, appears to have actually supported them in motivating their peers, as they mainly used the discussed water-promoting strategies. Of these, the influence agents mainly used face-to-face strategies and less often online strategies. In addition to applying the discussed water-promoting strategies, the influence agents also felt free to choose and devise their own strategies. This resulted in them also using more supportive strategies, such as providing support for the target behavior (“Can I fill your cup with water?”). They may have used these kind of face-to-face strategies more often because they fit more naturally into their usual peer-to-peer exchanges than online strategies [55].

In addition, our findings showed that the peers did not perceive that the influence agents had changed the descriptive norm concerning water drinking. However, there was a trend indicating that they did perceive that their peers thought that they should drink more water. This could be related to the finding that they also experienced more social support from their peers to drink water. A possible explanation for not finding any changes in the descriptive norm and for the trend for the injunctive norm may lie in the fact that the promotion of these norms must be made salient to achieve an effect [67]. However, the underlying approach of SNIs is that influence agents *informally* diffuse messages among their peers [28]. Therefore, in the training, the influence agents were taught to promote water using informal and non-salient strategies, such as drinking water themselves. This was done so that their peers would not notice that the agents were trying to influence their behavior and thus avoid reactance to the target health message [68].

### Intervention refinements

This study identified a number of possible refinements that could be made to *Share H*_*2*_*O* intervention. First, the influence agents did not succeed in increasing their peers’ intrinsic motivation and some of them even used strategies that could be considered as the opposite of autonomy-support—controlling strategies [69]—for instance, by turning it into a challenge and promising rewards. However, previous research has shown that intrinsic motivation, in particular, is a strong predictor of long-term changes in water consumption [37]. Therefore, the training activities could be improved by having a greater focus on teaching the influence agents to promote water drinking in a manner that fosters an intrinsically motivating environment for their peers. In relation to this, some influence agents also indicated that they had not sufficiently motivated their peers and had difficulty in doing so. Hence, another refinement in the content of influence agents training would be to provide more specific examples of how to promote water consumption but most importantly also practice real-life situations through role play [70]. To apply these refinements to the training and thus possibly make the intervention more effective, the contact moments could be extended. This could also contribute to the relatedness with the researchers and among the influence agents themselves [46].

Unexpectedly, some influence agents indicated that having a secret mission together was the most enjoyable aspect of the training. No part of the training was specifically developed with this intention but having a secret with others—thus group collaboration—may have motivated the influence agents to promote the behavior. By facilitating group collaboration, individuals experience feelings of belonging (i.e., the need for relatedness [45]), which may ultimately strengthen their intrinsic motivation [35]. Therefore, an avenue for refinement could be to emphasize group collaboration among influence agents, by focusing on the secret mission aspect, in order to motivate them to promote the target behavior. In addition, the additional analysis showed that the training approach effected the greatest change for boys and children in higher grade levels. It is therefore essential to make modifications to the training content so that it fits the entire target group. Nevertheless, it is important to note that there appeared to be no differences in changes for children from different levels of family affluence.

### Limitations and suggestions for future research

Some limitations should be addressed in interpreting the findings of this study. First, although the current paper collected data from the target group in the intervention (i.e., influence agents and their peers), it is important for future research to conduct a thorough process evaluation of the program, including data from other perspectives involved in the intervention, for example, from the trainers, teachers, and parents. Process evaluations consider factors beyond effectiveness to assess the implementation of the intervention, such as the intervention and theoretical fidelity, dose, reach, and context of the intervention. Examining these factors could help in understanding why a program was successful or not [71–73]. Related to this, in addition to the quantitative data, the current study only analyzed responses to open-ended questions to evaluate the implementation of the training. Therefore, we consider it important for future research to conduct interviews and focus groups with all parties involved in the SNI.

Third, the current study did not explicitly measure the extent to which the SDT-based techniques used in the training facilitated the psychological needs (i.e., autonomy, competence, and relatedness) [38]. It is therefore essential for future research to delve deeper into the process of these psychological mediators by including them as evaluation measures to explore the fidelity of the intervention to SDT (for an example, see [74]). Finally, the assessment of children’s beverage consumption was based on self-report. Although self-reported intakes with multiple 24-h recall measurements, including weekdays and weekend days, are generally considered reliable for children aged 4 to 11 years [75], one should keep in mind that there is the potential for under- or overreporting [76]. In addition, parents were not included as reporters to supplement the dietary intake information obtained from the children [75]. However, research has shown that children aged 10 years and older can reliably report their intake behavior [72]. Nevertheless, an interesting opportunity for future research would be to use an additional methodology, such as observations at school [77], and measure the beverage intake from different sources [78, 79].

## Conclusions

The findings of this study add important insights to the existing SNI literature by shedding light on how we can optimally motivate influence agents to engage in the target behavior and effectively support them in motivating their peers. The current study provides promising evidence for the use of an autonomy-supportive approach in the training of influence agents in SNIs. In particular, providing personally meaningful rationales for the target behavior, based on short-term benefits, seems to play an important role in motivating primary school children (i.e., aged 9 to 13 years old). Furthermore, for this age group, it seems important that SNIs focus on providing influencing agents with the skills to use face-to-face strategies, as well as giving them the freedom to choose how they wish to motivate their peers.

## Data Availability

The datasets used and/or analyzed in the current study are available from the corresponding author upon reasonable request.

## Abbreviations

SSB: Sugar-sweetened beverage
SNI: Social network intervention
SDT: Self-determination theory
T1: Baseline
T2: Immediately after the start of the intervention
M: Means
SD: Standard deviation
